# Retirement Age Modifies the Association Between Education/Occupation and Cognition

**DOI:** 10.1093/geroni/igab046.2658

**Published:** 2021-12-17

**Authors:** Monica Nelson, Ross Andel

**Affiliations:** 1 University of South Florida, Brandon, Florida, United States; 2 University of South Florida, Tampa, Florida, United States

## Abstract

Introduction: According to the cognitive reserve and use-it-or-lose-it hypotheses, engagement in stimulating activities seems to benefit cognition, with engagement often associated with more education or higher occupational position. However, whether retirement may modify the association between education/occupation and cognition is unclear. We aimed to assess how age at retirement may modify the relationship between education/occupation and cognition. Methods: Older adults (n=360) from the Alzheimer’s Disease Neuroimaging Initiative who were cognitively normal and retired at baseline participated. Linear regression was used to assess how educational attainment (high/low) or occupational position (managerial, intermediate/clerical, routine/manual) related to executive functioning (EF) or memory, controlling for age, sex, depressive symptoms, and health status. Effect modification by retirement (early, on-time, late). Results: High education (EF: b=0.37, SE=0.08, p<.001; memory: b=0.22, SE=0.05, p<.001), intermediate (EF: b=0.26, SE=0.11, p=.019; memory: b=0.18, SE=0.08, p=.018) and managerial (EF: b=0.23, SE=0.12, p=.045; memory: b=0.16, SE=0.08, p=.045) occupations (compared to routine/manual occupations) were associated with better EF and memory performance. High education was significantly associated with better EF and memory for participants who retired early (EF: b=0.43, SE=0.12, p<.001; memory: b=0.29, SE=0.10, p=.004) or on-time (EF: b=0.51, SE=0.15, p=.001; memory: b=0.24, SE=0.10, p=.014), but not for participants who retired late (EF: b=0.19, SE=0.15, p=.200; memory: b=0.09, SE=0.09, p=.334). Intermediate occupations were associated with EF only for participants who retired on-time (b=0.58, SE=0.21, p=.007). Conclusion: Education and occupational position may influence cognition after retirement differently based on retirement timing, with effects most apparent for on-time retirement and substantially reduced for late retirement.

